# High cell density fed-batch production of insecticidal recombinant ribotoxin hirsutellin A from *Pichia pastoris*

**DOI:** 10.1186/s12934-018-0992-x

**Published:** 2018-09-14

**Authors:** Hongbo Li, Yuxian Xia

**Affiliations:** 1 Postdoctoral Mobile Station of Biology, Genetic Engineering Research Center, College of Life Sciences, Chongqing University, Chongqing, 400030 China; 2 College of Biological and Food Engineering, Huaihua University, Huaihua, 418008 China; 3 Genetic Engineering Research Center, College of Life Sciences, Chongqing University, No. 55 South Road of University Town, Shapingba District, Chongqing, 401331 China

**Keywords:** Ribotoxin, Hirsutellin A, *Pichia pastoris*, Fed-batch, Purification

## Abstract

**Background:**

The fungal ribotoxin hirsutellin A (HtA) exhibits strong insecticidal activity; however, efficient systems for expressing recombinant HtA (rHtA) are lacking. Here, we established an efficient heterologous expression system to produce large amounts of rHtA.

**Results:**

Recombinant *Pichia pastoris* transformants with high levels of secretory rHtA were screened, and in a fed-batch reactor, rHtA was secreted at levels up to 80 mg/l following methanol induction, which was more than sixfold higher than that in shake flasks. Approximately 7 mg of highly pure rHtA was obtained from 300 ml of fed-batch culture supernatant by Ni^+^-nitriloacetic acid affinity chromatography and CM Sepharose ion-exchange chromatography. Mass spectrometry results revealed rHtA as a native N-terminal non-glycosylated monomeric protein with a molecular weight of 15.3 kDa. Purified rHtA exhibited excellent thermal and protease stability and dose-dependent cytotoxicity to Sf9 insect cells and insecticidal activity against *Galleria mellonella* larvae.

**Conclusions:**

This is the first report of rHtA expression in *P. pastoris.* The rHtA was expressed at a high level under high-cell-density fed-batch fermentation and was efficiently purified using a two-step purification method. Purified rHtA exhibited thermal and protease stability, as well as appropriate bioactivities. Our results indicate that fed-batch production by *P. pastoris* is an efficient method to produce functional rHtA.

**Electronic supplementary material:**

The online version of this article (10.1186/s12934-018-0992-x) contains supplementary material, which is available to authorized users.

## Background

Chemical pesticides are widely used. However, the extensive use of these pesticides also affects non-target insects and animals, thereby impairing ecosystem, potentially posing a risk to human health, and causing diseases, including cancer, reproductive disorders, neurological disorders, and allergies. Therefore, there is a consensus to reduce the use of pesticides [1]. To better protect the ecological environment and human health, and to maintain sustainable agricultural development, it is urgent to develop “green” pesticides [2–5]. Fungi are the major source of biological pesticides [6–8]. Their metabolites, including toxic proteins and peptides, have the potential to be used in biological pest control [9–11].

Ribotoxins produced by some fungal species, such as *Aspergillus*, *Hirsutella thompsonii* and other entomopathogenic fungi, have the potential to be used as biological pesticides [10, 12, 13]. Specifically, the fungal ribotoxin hirsutellin A (HtA) produced by the invertebrate fungal pathogen *H. thompsonii* exhibits insect-specific cytotoxicity and strong insecticidal properties [14, 15]. Native HtA is a non-glycosylated monomeric protein comprising 130 amino acid residues and shows thermo stability and protease stability. As a comparison, HtA is 10–20 amino acids shorter than ribotoxins from *Aspergillus*, and has a low homology with them (25%), which is the main reason leading to significant differences in their biological functions [14, 16, 17]. Previous studies have reported that HtA is highly toxic to adult mites and aphids, lethal to moth and fly larvae, and shows oral toxicity to neonatal larvae of *Aedes aegypti* [14, 15]. However, the content of native HtA is low, with only 35 μg HtA isolated from the supernatant of 1 g of dried mycelium and less than 1 mg HtA purified from 1 l of fermentation broth [14]. Although recombinant HtA (rHtA) has been successfully prepared using *Escherichia coli* (*E. coli*) expression system, less than 1 mg rHtA could be purified from 1 l of culture medium using a complex purification protocol [12]. Moreover, *E. coli*-expressed rHtA is not the N-terminal native protein, resulting in potentially different biological activity. Additionally, *E. coli* produces large amounts of endotoxin, which needs to be removed before in vivo activity analyses. The failure to prepare large amounts of HtA has seriously limited the further development of its insecticidal potential.

Investigation of HtA bioactivity against insect pests requires large quantities of protein [14, 15]. In particular, the determination of the oral insecticidal activity of HtA against agricultural pests and its biological safety to mammals also requires a large amount of protein [14, 15]. Therefore, it is necessary to develop a heterologous protein expression system and efficient purification method to prepare a large amount of active rHtA. As a widely used high-level eukaryotic protein-expression system, *Pichia pastoris* (*P. pastoris*) has the ability to secrete recombinant proteins [18]. Additionally, *P. pastoris* has the ability to produce gram-level amounts of secretory recombinant protein per litre of fermentation culture [19]. Furthermore, *P. pastoris* does not produce endotoxin. Therefore, purified recombinant proteins can be directly used for in vivo experiments.

In this study, we reported a method for efficient expression and purification of rHtA from *P. pastoris* X33 by fed-batch fermentation. Also, we analysed the bioactivity of rHtA.

## Results

### Plasmid construction and selection of *P. pastoris* transformants

**Fig. 1:**
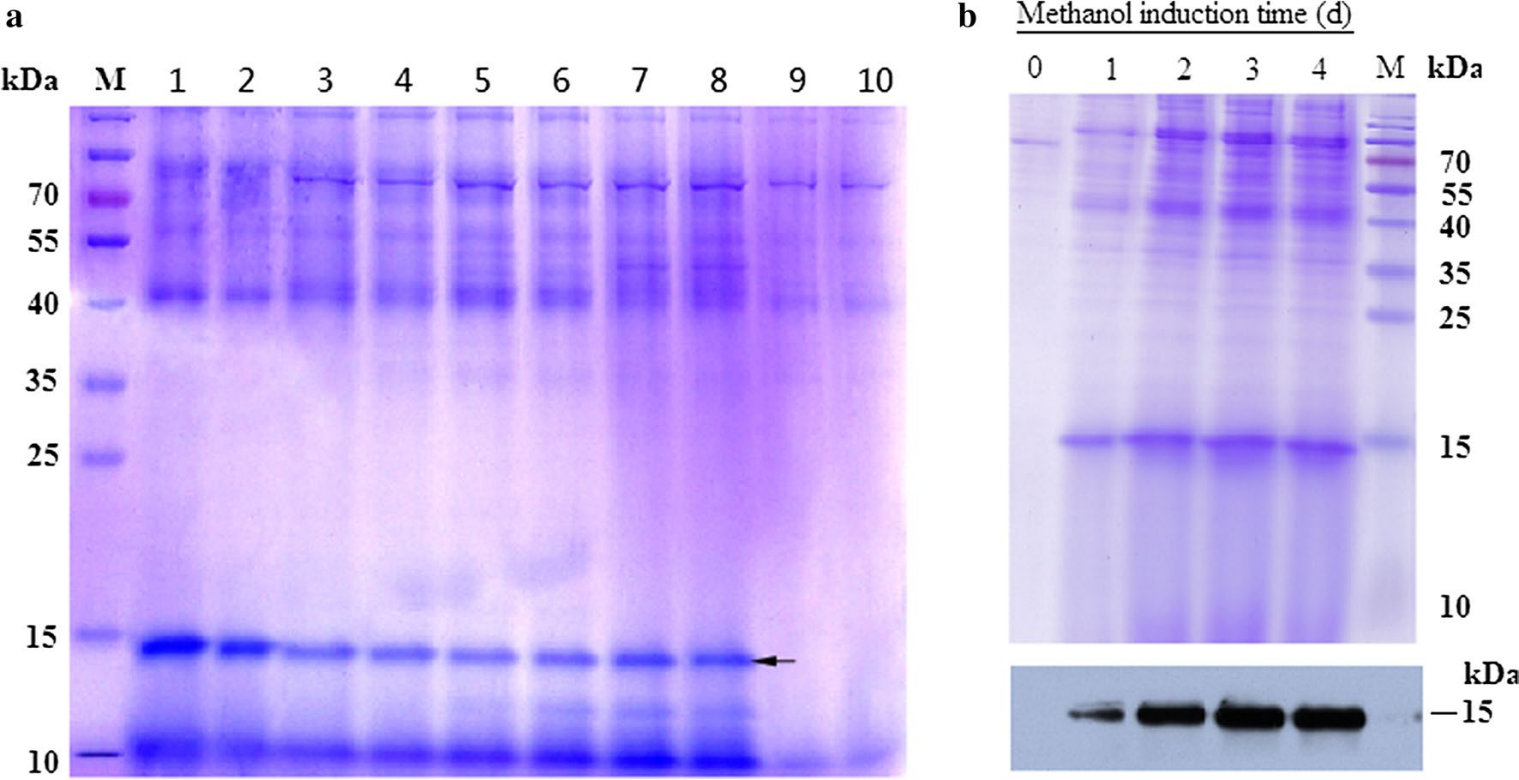
Detection of rHtA expression. **a** SDS-PAGE analysis of secreted rHtA transformants. Lanes 1–8, BMMY culture supernatant of selected transformants from 1 g/l zeocin YPD plates; lane 9, BMMY culture supernatant of the untransformed X33 strain; lane 10, BMMY culture supernatant of empty vector transformants; lane M, protein marker. The expressed rHtA was marked with an arrow. **b** SDS-PAGE and western blot analysis of rHtA. Culture supernatant was collected at the indicated time (0, 1, 2, 3 and 4 days) after methanol induction in flasks. Precipitation was initiated using final concentration of 10% TCA, and proteins were analysed by 15% SDS-PAGE and stained with Coomassie Brilliant Blue R250. Lane M, protein marker

Following a 72-h incubation at 28 °C, single colonies from YPD plates containing 1 mg/ml zeocin were picked and amplified by PCR. A fragment of ~ 400 bp was generated, suggesting integration of the pPICZαA-*HtA* plasmid into the *P. pastoris* genome (data not shown). Based on the amino acid sequence of rHtA, the theoretical MW of rHtA was ~ 15 kDa according to Expasy prediction (http://www.expasy.ch/cgi-bin/pi_tool). SDS-PAGE results for all screened transformants (Fig. 1a) indicated their ability to secrete a protein with a MW similar to that predicted for rHtA, whereas control transformants (pPICZαA) and samples from the untransformed X33 strain showed no visible protein band at the predicted MW. Among these transformants, the transformant in Lane 1 had the highest level of rHtA expression and fewer contaminated proteins. Thus, this transformant was used for the following high cell density fermentation. These results showed that the pPICZαA-HtA plasmid was successfully constructed and could produce secreted rHtA in the *P. pastoris* X33 strain.

### Time course of rHtA expression in shake flasks

We detected a protein at ~ 15 kDa by SDS-PAGE following a 1-day methanol induction (Fig. 1b). The protein content reached a maximum level at 3 days of methanol induction. However, the proportion of rHtA did not significantly increase along with increased induction time after 3 days. At 3-days post-induction, the band for rHtA equated to ~ 3 μg, suggesting that the concentration of secreted rHtA was ~ 13 mg/l in the media. Western blot results confirmed that the recombinant protein was rHtA, and that the highest level of rHtA was obtained after 3 days of methanol induction (Fig. 1b).

### Fed-batch fermentation

**Fig. 2:**
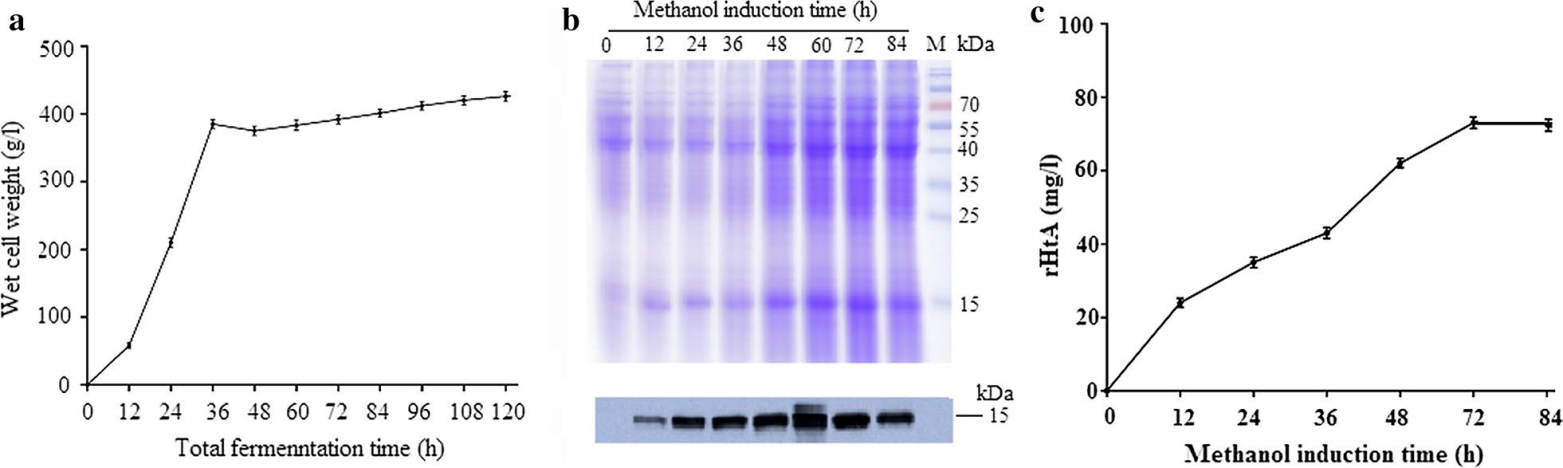
Analysis of rHtA expression under fermentation conditions. **a** Time course of changes in wet-cell weight in a 5-l bioreactor. Cells were collected at the indicated times. The wet weight of cultured cells was obtained by centrifugation at 16000*g* for 15 min at 4 °C. **b** SDS-PAGE analysis of the time course associated with rHtA expression in the 5-l bioreactor. Culture supernatant was collected at the indicated times after methanol induction. Protein concentration in the supernatant was determined by Bradford assay, and BSA was used as the standard. **c** Detection of rHtA concentration by gel scanning. Culture supernatant was collected at the indicated times after methanol induction. The supernatants were precipitated, then analyzed on 15% SDS-PAGE and stained with Coomassie blue R250. After the decolorization, the protein imaging system scanned the SDS-PAGE gel. The gray scale of the protein strip was analyzed by Quantity One software (Bio-Rad) and the concentrations of target bands were calculated

Glycerol fed-batch culture was initiated after the initial glycerol was exhausted based on increased dissolved oxygen (DO) levels and decreased stirring. During this phase of biomass increase, highest biomass level was achieved at a feed-solution flow rate of 24 ml/h, with the wet weight of *P. pastoris* cells reaching 390 g/l before the addition of methanol. Within 12 h after the addition of methanol, cell density declined, indicating that the change in carbon source significantly inhibited the growth of yeast. With the adaptation of *P. pastoris* to methanol, the yeast began to grow slowly, and the wet weight of the cells reached ~ 440 g/l by the end of fermentation (Fig. 2a). During fed-batch fermentation, rHtA secretion was detected at 12-h post-methanol induction, with secreted rHtA reaching a peak at ~ 60 to ~ 72 h after the addition of methanol. However, prolonging the induction time resulted in decreases in rHtA content (Fig. 2b). Western blot results confirmed that that the highest rHtA concentration was obtained at 72-h post-induction. Gel-scanning results indicated an rHtA concentration of ~ 78 mg/l in fed-batch supernatant at 72-h post-induction (Fig. 2c), which was more than sixfold higher than that in shake flasks (~ 13 mg/l; Fig. 1b). These results demonstrate that high cell-density culture of *P. pastoris* offers high productivity of rHtA in the bioreactor.

### Purification of rHtA

**Fig. 3:**
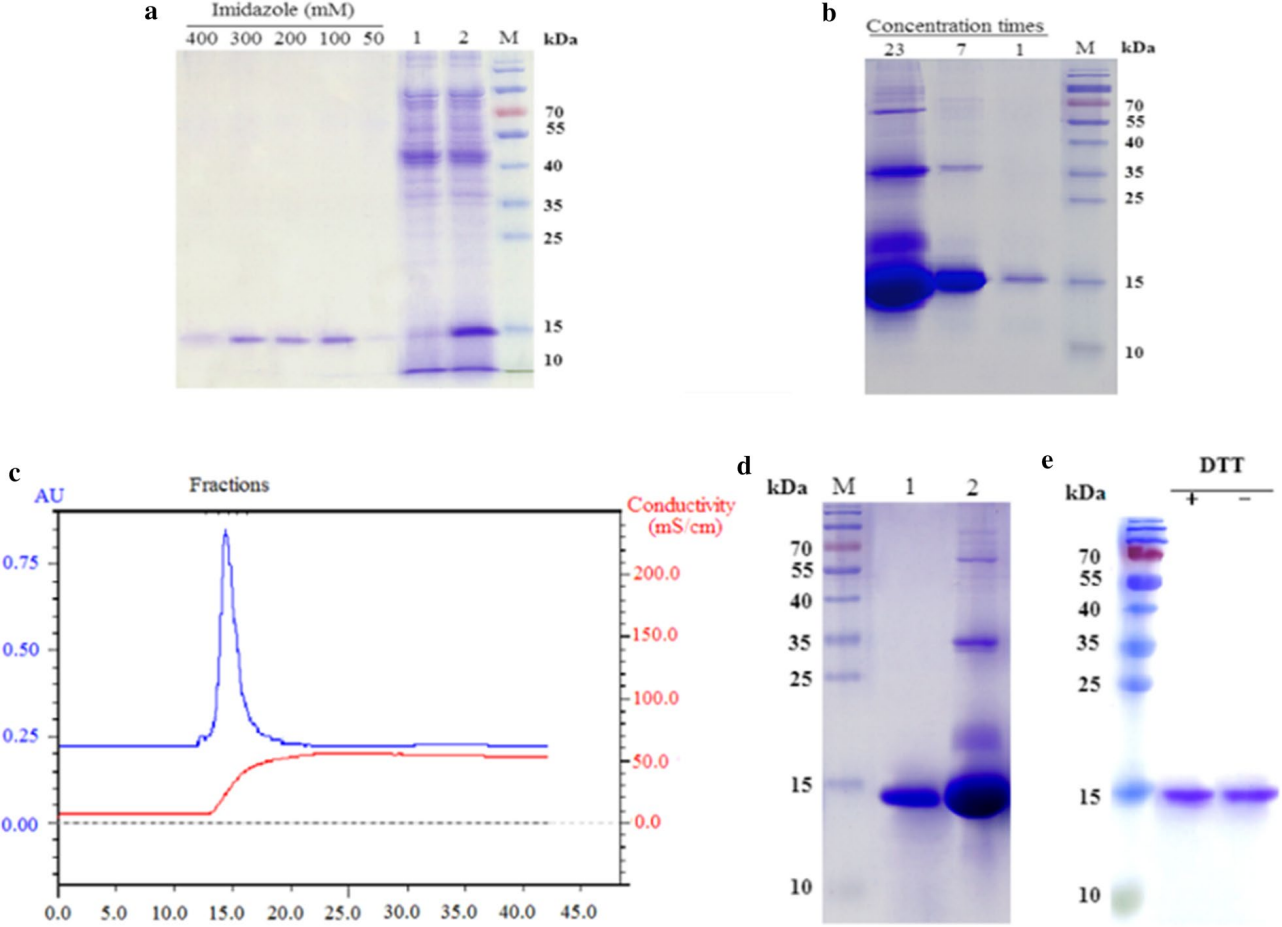
Purification of rHtA. **a** Purification of rHtA by Ni^+^-NTA chromatography. Lane 1, elution sample following loading; lane 2, elution prior to purification. **b** Analysis of concentrated rHtA purity following Ni^+^-NTA purification. Lane 23, 23-fold concentration; lane 7, sevenfold concentration; lane 8, eluted rHtA (unconcentrated); lane M, protein marker. **c** Elution curves of rHtA by ion-exchange chromatography. The eluents were collected according to OD_280nm_ > 250 mAU. The blue curve and the red curve represent the UV absorbance (mAU) and ionic strength (mS/cm) of the eluent, respectively. **d** SDS-PAGE analysis (15%) of rHtA by ion-exchange chromatography. Lane 1, eluted rHtA; lane 2, 23-fold concentration of the eluent from the Ni^+^-NTA column; lane M, protein marker. **e** SDS-PAGE analysis of concentrated rHtA by ion-exchange chromatography under reducing and non-reducing conditions. Lane 1, reduced conditions; lane 2, non-reducing conditions; lane M, protein marker

Recombinant HtA was able to bind to Ni^+^-NTA resin (Fig. 3a). After washing with buffer containing 30 mM imidazole, protein contaminants were removed, and at 50 mM imidazole, small amounts of rHtA began to elute, with rHtA elution increasing along with imidazole concentrations up to 500 mM. Approximately 12.3 mg rHtA was obtained from 300-ml fed-batch supernatant by Ni^+^-NTA affinity purification, equivalent to a ~ 61% recovery rate. The eluted samples from the Ni^+^-NTA column showed a single protein band, whereas contaminated proteins became visible following concentration of the samples by sevenfold (Fig. 3b). After dialysis, the rHtA solution was subjected to CM ion-exchange chromatography (Fig. 3c), resulting in a single protein peak, following elimination of the contaminating proteins (Fig. 3d). Following this step, acquisition of ~ 7 mg pure rHtA with a ~ 34% recovery rate was yielded from 300 ml of crude culture supernatant, with a purity of up to 95% (Table 1). Under both reducing and non-reducing conditions, SDS-PAGE analysis revealed a single band for purified rHtA (Fig. 3e), indicating that rHtA was a monomeric protein expressed in *P. pastoris*. These results indicate that high purity rHtA could be prepared by Ni^+^-NTA affinity purification and CM ion-exchange chromatography.

**Table 1:** Purification of rHtA from the fed-batch ferment supernatant

Purification steps	Total volume (ml)	rHtA (mg)	Recovery (%)
Supernatant before purification	300	20.2	100
Ni^+^ affinity chromatography	100	12.3	61
CM chromatography	4.1	6.9	34

### Determination of rHtA MW and amino acid sequence

**Fig. 4:**
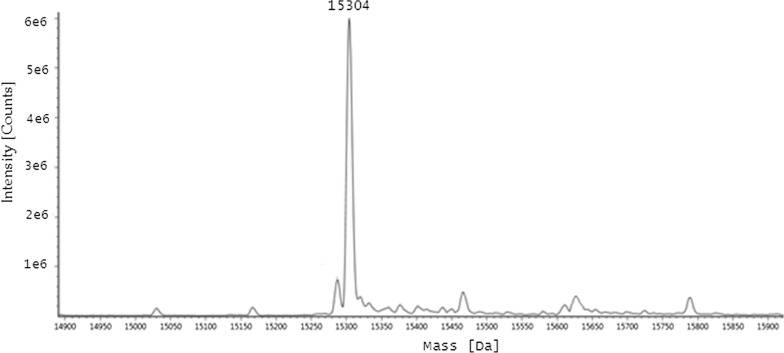
MS analysis of rHtA. Purified rHtA was measured By MS. The MW of rHtA was calculated at ~ 15.3 kDa

MW determination by Q-TOF–MS showed that the average MW of purified rHtA was 15.304 kDa (Fig. 4), which was similar to the theoretical MW (~ 15.308 kDa), suggesting that purified rHtA from *P. pastoris* is a non-glycosylated protein. Nano-LC–MS/MS results identified 34 peptides (Table 2) matching the 136 amino acids in the sequence of full-length rHtA. LC–MS/MS results verified that purified rHtA harboured a native N-terminus and a fused 6× His tag at the C-terminus.

**Table 2:** Peptide segments were observed by nanoLC–MS/MS

No.	Sequence	No.	Sequence	No.	Sequence
1	APIVTCR	13	SGDPHRYFAGDHIR	25	GGPTPIR
2	APIVTCRPK	14	YFAGDHIR	26	GGPTPIRVVYANSR
3	APIVTCRPKLDGR	15	YFAGDHIRWGVNNCDK	27	VVYANSR
4	LDGREKPFK	16	WGVNNCDK	28	GAVQYCGVMTHSK
5	EKPFKVDVATAQAQAR	17	WGVNNCDKADAILWEYPIYWVGK	29	GAVQYCGVMTHSKVDK
6	PFKVDVATAQAQAR	18	ADAILWEYPIYWVGK	30	GAVQYCGVMTHSKVDKNNQGK
7	VDVATAQAQAR	19	ADAILWEYPIYWVGKNAEWAK	31	VDKNNQGK
8	VDVATAQAQARK	20	NAEWAKDVK	32	VDKNNQGKEFFEK
9	KAGLTTGK	21	NAEWAKDVKTSQQK	33	NNQGKEFFEK
10	KAGLTTGKSGDPHR	22	DVKTSQQK	34	EFFEKCDHHHHHH
11	AGLTTGK	23	DVKTSQQKGGPTPIR		
12	AGLTTGKSGDPHR	24	TSQQKGGPTPIR		

### Analysis of the thermal- and protease-stability of the purified rHtA

**Fig. 5:**
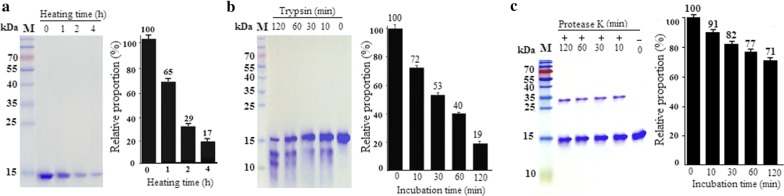
Thermal- and protease-stability analyses. **a** Thermal-stability analysis. **b** Proteinase K-stability analysis. **c** Trypsin-stability analysis. Purified rHtA was incubated at the indicated times

The results of SDS-PAGE and thermal- and protease-stability studies are shown in Fig. 5. The protein became insoluble after thermal-denaturation treatment, with the denatured proteins precipitating during high-speed centrifugation. After 30-min incubation at 98 °C, 65% of rHtA remained soluble and nearly 20% of rHtA remained soluble after 4 h of heat incubation (Fig. 5a). These results showed that purified rHtA showed high thermal tolerance.

Protease-stability studies showed that 28% of rHtA underwent digestion after a 10-min incubation with trypsin, and 53% of rHtA was digested after 30 min. Prolonged digestion of 2 h resulted in > 20% of purified rHtA remaining intact (Fig. 5b). Protease treatment using proteinase K showed that rHtA exhibited excellent stability, with only 10% of rHtA digested after 10 min and 30% digested after a 2-h digestion (Fig. 5c). These results indicate that rHtA is more stable in the presence of proteinase K and exhibits overall excellent stability to protease treatment.

### The purified rHtA inhibits the growth of sf9 insect cells

**Fig. 6:**
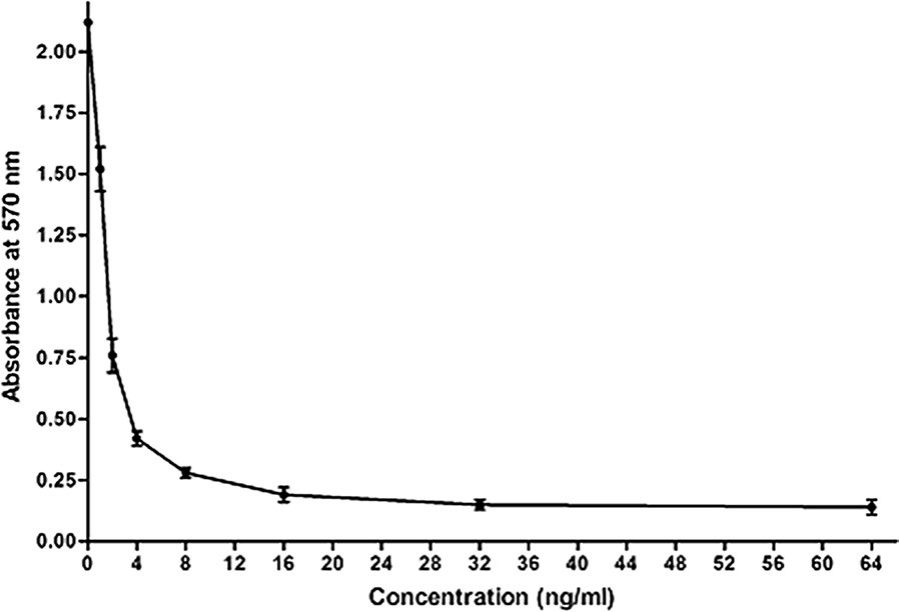
MTT assay of Sf9 cell proliferation. The growth of Sf9 cells was analysed at the given concentrations of rHtA in 96-well plates. The X-axis shows the absorbance at 570 nm, and the Y-axis shows the final rHtA concentration added to the culture medium. Data represent the mean ± standard deviation of triplicate experiments

Effect of purified rHtA on cell proliferation of sf9 insect cells was analyzed by MTT assay. Without adding rHtA to the media, the absorbance value of MTT at 570 nm was 2.3. After addition of 1 ng/ml rHtA to insect-cell medium, the absorbance value of MTT at 570 nm was about 1.6, which means 70% of the cells remained viable as compared with those treated with PBS (Fig. 6). When the final rHtA concentration was increased to 2 ng/ml, about 35% of the cells remained viable relative to controls. Further increases in rHtA concentration (4–16 ng/ml) in the media resulted in continued decreases in cell viability. When the rHtA concentration was over 32 ng/ml, few viable cells remained. These results confirmed the ability of rHtA to inhibit the growth of sf9 insect cells, demonstrating that rHtA purified from *P. pastoris* has cytotoxic effects on sf9 cells.

### The purified rHtA is toxic to *G. mellonella*

**Fig. 7:**
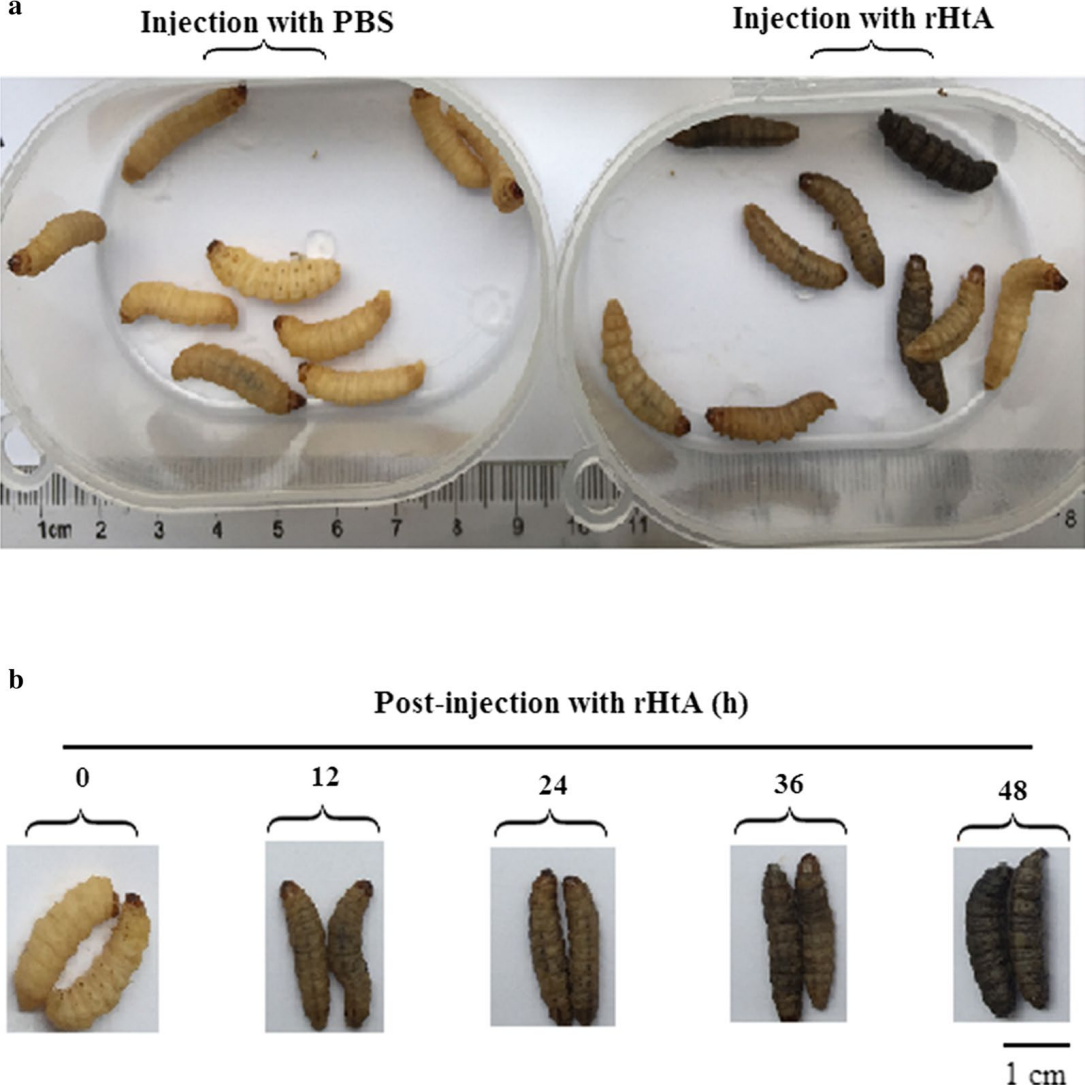
Injection-toxicity assays using rHtA. **a** *Galleria mellonella* larvae injected with 4 μg of purified rHtA or PBS as a control. **b** Time course of *G. mellonella* larvae injected with 4 μg of purified rHtA per larvae

*Galleria mellonella* injected with rHtA showed classic symptoms of ribotoxin poisoning, including brownish coloration progressing to black upon death, whereas no discoloration was observed in worms injected with PBS (Fig. 7a). Insect-toxicity results are shown in Table 3. Upon increasing the dose to 1 μg, 6 larvae (20%) died after 3 days, and the dead larvae were black. Different injection times showed obvious differences in associated mortality, and the dose of 4 μg resulted in 100% mortality after 4 days (Fig. 7b). Discoloration at this dose began at 12 h and darker shading was observed after 24 h. And, death was observed after 36 h. The dose of 8 μg resulted in 100% mortality after 3 days. Although 6.7% of control *G. mellonella* larvae died after 4 days, their coloration had not changed, suggesting no symptoms of ribotoxin poisoning. These results indicate that rHtA is toxic to *G. mellonella.*

**Table 3:** Injection toxicity assays of rHtA

Samples (μg/larvae)	Mortality (%)				
	1 day post injection	2 days post injection	3 days post injection	4 days post injection	5 days post injection
PBS	0	0	0	6.7	6.7
1	0	9.9	20.0	26.7	40.0
2	13.3	33.3	46.7	66.7	93.3
4	36.7	83.3	93.3	100	100
8	53.3	90	100	100	100

## Discussion

More than 20 years since the discovery of HtA, there has not been any report on its application in biological control [14, 15]. The most likely reason is that an efficient heterologous expression system has not yet been established, leading to the uncertainty of its insecticidal activity against agricultural pests. In this study, a large amount of rHtA was prepared from *P. pastoris* by high density fermentation. Compared with the shaking flasks fermentation in BMMY medium, the fed-batch high cell density fermentation of *P. pastoris* in basic salt media produced significantly higher rHtA. DO is critical to expression efficiency in the *P. pastoris* system [20, 21]. Heat transfer is another important limiting factor in high cell density *P. pastoris* cultivations [22]. The NCBIO bioreactor system is able to maintain constant temperature and 30% DO by feeding and DO-series connection. As a result, as high as about 440 g/l wet weight of *P. pastoris* cells were produced by the end of culture in the NCBIO bioreactor. In previous reports, several grams per litre of recombinant proteins have been produced during fed-batch production processes [23, 24]. In this study, though the protein was not obtained at gram per litre level, it is enough to produce rHtA for the insecticidal activity analysis, especially for its oral insecticidal activity against agricultural pests. And, the safety of HtA, which cannot be performed without a large amount of purified rHtA protein, may be comprehensively tested. Additionally, the establishment of rHtA efficient preparation system will also facilitate us to carry out the safety tests in mammalians.

Gene-copy number is an important factor affecting the expression of recombinant proteins, and the most common strategy for selecting *P. pastoris* transformants is to screen multi-copy integrations of an expression cassette [25–27]. In this study, transformants from YPD plates treated with 1 mg/ml zeocin were selected. Almost all the selected transformants showed high levels of rHtA secretion, suggesting that large number of expression cassettes are incorporated into *P. pastoris*. Colonies obtained using LiCl were limited, with only 20 colonies selected from 1 mg/ml zeocin YPD plates, and no transformants obtained from plates with higher zeocin concentration (1.5 mg/ml zeocin).

All published protocols related to rHtA purification consist of at least four different purification steps to obtain > 95% purity [15]. In the present study, ~ 7 mg of highly pure rHtA was obtained using a two-step purification procedure from 300 ml of crude fermentation supernatant. The concentration of purified rHtA was > 20-fold higher that that previously reported in *E. coli* [12]. This purification procedure resulted in higher levels of purified rHtA with fewer contaminated proteins. Although nickel-affinity chromatography eliminated most of the contaminated proteins, additional round of chromatographic purification were necessary. Additional purification steps involving dialysis using a buffer with a higher pH eliminated the binding of contaminated proteins to the Sepharose column. Here, the purified rHtA variant contained a 6× His tag at the C-terminus. In previous studies, rhPDGF-AA with a native amino acid sequence was purified from *P. pastoris* by one-step CM ion-exchange chromatography at its theoretical pI of 8.6 [28]. The theoretical pI of rHtA is 9.3, which is higher than that of rhPDGF-AA, indicating that native rHtA might be capable of purification using a single step of ion-exchange chromatography directly from the *P. pastoris* fermentation supernatant. Glycosylation is a common posttranslational protein modification in *P. pastoris,* which is frequently found on secreted proteins [18, 19]. Here, determination of rHtA MW indicated that the purified variant was not glycosylated. Therefore, these results suggest that *P. pastoris* is suitable for the preparation of large amounts of unmodified and highly active rHtA.

The thermo stability of insecticidal protein is an important factor affecting its application in biological control. In this study, the thermal stability of rHtA was assayed, revealing that almost all rHtA remained soluble following incubation at 56 °C for 24 h (data not shown). After incubation at 98 °C for 1 h, 65% rHtA remained intact, which is consistent with a previous report [14]. The soluble rHtA after heat treatment remained bioactivity by injecting *G. mellonella* larvae (data not shown). High thermal stability might facilitate the use of rHtA as an insecticide.

The stability of rHtA to protease also affects its insecticidal activity. In this study, rHtA was not sensitive to protein K. However, protease-stability analysis showed two degradation bands following trypsin digestion, with each band having an MW between 10 and 15 kDa. In this study, the rHtA solution treated by proteases also remained bioactivity by injecting *G. mellonella* larvae (data not shown). A previous report indicated that trypsin digestion at room temperature overnight did not affect the insecticidal activity of native HtA, indicating that the protein fragments digested by trypsin may be still biologically active [14]. The three-dimensional structure of HtA also suggests that maintenance of ribosomal-toxin activity does not require the full-length sequence [12]. Therefore, further study of the trypsin-digested fragments might help determine the structural components necessary for HtA activity.

In vivo injection of native HtA is highly toxic to insects, such as *G. mellonella* larvae [29]. In the present study, injection of 1 μg rHtA per larvae, equivalent to 4 μg/g body weight, resulted in 40% mortality after 5 days, which is similar to results using native HtA (mortality after 8.8 days) [14]. Additionally, *G. mellonella* larval activity was obviously weaker following injection with rHtA relative to controls (Additional file 1). These results indicate that rHtA expressed in *P. pastoris* shows comparable activity as that of native HtA. HtA exhibits strong activity following injection and obvious oral toxicity, possibly also exhibiting contact activity, which may represent an insecticidal pathway rare among other insecticidal proteins [14]. High levels of insecticidal activity and multiple insecticidal pathways expand the potential applications of such proteins in biological control. Future studies on the oral and/or contact toxicity of HtA are warranted.

Some insecticidal proteins, such as insect neurotoxins, exhibit strong haemocoel-injection activity and can be used to enhance the insecticidal activity of recombinant proteins produced by microorganisms, such as insect pathogenic viruses and entomopathogenic fungi [30–32]. Some insecticidal proteins result in oral toxicity and can be used to obtain genetic engineered plants for insect control [33, 34]. HtA is cytotoxic to insect cells and mammalian cells, suggesting it as an indiscriminate insecticidal protein, which might limit its potential use as an insecticidal agent. Fortunately, the potential toxicity of HtA against vertebrates can be overcome by the design of new variants, such as those involving Trp mutations [35]. Protein fusions can also enhance insecticidal activity or alter the insecticidal pathway [36, 37]. Therefore, HtA might be a candidate for genetic engineering and may fuse with other insecticidal proteins, such as snowdrop lectin, specific insect neurotoxins (from spider, scorpion or other poisonous animals) or Cry toxin from *Bacillus thuringiensis*, to enhance its insecticidal activity or change its insecticidal pathway.

## Conclusions

In conclusion, for the first time, this study reported high levels of rHtA expression by high-cell-density fed-batch fermentation in *P. pastoris*. Highly pure rHtA was obtained using a two-step purification method. The steps were simpler and more efficient than previously reported purification protocols. Protein assays showed that rHtA was a non-glycosylated monomeric protein with a native N-terminal region, and had excellent thermal and protease stability and high levels of insecticidal activities. The preparation of large amounts of highly active rHtA will facilitate insecticidal-activity studies on agricultural pests and contribute to the development of new bioinsecticide.

## Methods

### Construction of expression plasmid and transformation of *P. pastoris*

*HtA* harbouring a 6× His tag at the C-terminus was synthesized according to its amino acid sequence and *P. pastoris* codon bias (http://www.kazusa.or.jp/codon/cgi-bin/showcodon.cgi?species=4922). To obtain native N-terminal rHtA, a Kex2 signal-cleavage site (aaa aga) was fused to the upstream region of the synthetic cDNA. The sequence of the synthetic *HtA* gene was as follows: *CTCGAG* aaa aga GCCCCAATCGTCACCTGCAGACCAAAGTTGGACGGTAGAGAGAAGCCATTCAAGGTCGACGTCGCCACTGCCCAGGCTCAAGCCAGAAAGGCCGGTTTGACCACCGGTAAGTCCGGTGACCCACACAGATACTTCGCCGGAGACCACATCAGATGGGGTGTCAACAACTGCGACAAGGCCGACGCCATCTTGTGGGAGTACCCAATCTACTGGGTCGGTAAGAACGCCGAGTGGGCCAAGGACGTCAAGACCTCCCAGCAGAAGGGTGGACCAACCCCAATCAGAGTTGTCTACGCCAACTCCAGAGGTGCCGTCCAATACTGCGGAGTCATGACCCACTCCAAGGTCGACAAGAACAACCAGGGAAAGGAGTTCTTCGAGAAGTGCGACCACCACCACCACCACCAC TAA *TCTAGA*. The synthesized DNA fragments of *HtA* were digested with *Xho*I and *Xba*I and inserted into the pPICZαA vector (Invitrogen, Carlsbad, CA, USA). The expression vector was linearized by digestion with *Sac*I and transformed into *P. pastoris* X33 using LiCl according to manufacturer instructions (Invitrogen, Carlsbad, CA, USA). *P. pastoris* transformants were grown on yeast extract-peptone-dextrose (YPD) plates containing 100 μg/ml zeocin. Transformants with multiple expression cassettes were screened using increasing concentrations of zeocin from 200, 400, 800, and 1000 μg/ml. Positive transformants were verified by PCR.

### Screening of transformants with high level expression of rHtA

After incubation at 28 °C for 3 days on the YPD plates containing 1000 μg/ml zeocin, twenty positive transformants verified by PCR were grown respectively in 10 ml buffered-glycerol complex medium (BMGY) at 28 °C with shaking at 250 rpm for 24 h. Cells were harvested by centrifugation at 1500*g* for 10 min at 28 °C and resuspended in 2 ml buffered-methanol complex medium (BMMY). Twenty microliters of 100% methanol was added to each tube every 24 h. After induction at 28 °C with shaking at 250 rpm for 72 h, cell cultures were collected by centrifugation at 12,000*g* for 10 min at 4 °C. Supernatants (0.9 ml) were mixed with 0.1 ml 100% trichloroacetic acid (TCA) and incubated at − 20 °C for 4 h. After centrifugation at 12,000*g* for 30 min at 4 °C, precipitates were washed with 1 ml acetone by centrifugation at 12,000*g* for 10 min at 4 °C, followed by resuspension in 50 μl of 1× reduced loading buffer containing 8 M urea. Samples (10 μl) were analysed by SDS-PAGE.

### Time course of rHtA expression in shake flasks

The colony with the highest rHtA expression was cultured in 400 ml BMGY with shaking at 28 °C to an optical density at 600 nm of ~ 15. Cells were harvested by centrifugation at 1500*g* for 10 min at 28 °C, followed by resuspension in 60 ml BMMY and growth for 4 days at 28 °C with shaking at 250 rpm. Each day, 600 μl of 100% methanol was added to the medium. Before adding methanol, 2 ml culture samples were collected by centrifugation at 15,000*g* for 10 min at 4 °C, and 0.9 ml samples of the supernatants were precipitated with TCA according to methods described in the previous section. The pellets were resuspended in 40 μl of 1× SDS loading buffer containing 8 M urea and subjected to SDS-PAGE analysis. The amount of secreted rHtA was determined by scanning the gel using Quantity One software (Bio-Rad, Hercules, CA, USA). The time course associated with rHtA expression was confirmed by western blot analysis using a rabbit anti-His×6 IgG antibody (CST, Guangzhou, China).

### Fed-batch fermentation

One colony with high rHtA expression was used to inoculate into 5 ml YPD media, grown for 20 h at 28 °C, followed by scaling up with 300 ml fed-batch basal-salts media containing 2% (w/v) glycerol and shaking at 250 rpm and 28 °C until reaching an optical density at 600 nm of 10. Scale-up expression was performed in a 5-l bioreactor (NCBIO, China, Shanghai) using 2.7-l basal-salts medium. The composition and preparation of fed-batch basal-salts medium and trace metal solution (TMS) were referred to previous reports [38]. Feeding mixture contains: 100 ml of 70% glycerol solution plus 1.2 ml TMS. Induction mixture contains: 100 ml of methanol plus 1.2 ml TMS. Compressed air was maintained at 6 l/min, dissolved oxygen (DO) was maintained at > 40% saturation by stirring at between 200 and 1000 rpm, and temperature was maintained at 28 °C. Fermentation was initiated by adding 300 ml of fed-batch basal-salts seed culture into 2.7-l basal-salts medium, with the medium pH maintained at 6.0 by adding 30% ammonium hydroxide. To maintain glycerol concentration in the glycerol fed-batch phase, the feeding mixture was added automatically according to DO > 40% saturation by automatic series regulation. After all feed solution was consumed, the induction solution was added to initiate an 84-h induction, and the medium pH was maintained at 6.0 by adding 30% ammonium hydroxide. The methanol-feeding method used for induction was as follows: the feeding rate of methanol was controlled at 3.6 ml/h for 12 h, 5.4 ml/h for 24 h, 7.2 ml/h for 36 h, and 5.4 ml/h for 12 h. Cell-culture samples (10 ml) were collected at 0, 12, 24, 36, 48, 60, 72, and 84 h after methanol induction. For each time-point, 0.9 ml of supernatant was precipitated with TCA as described, precipitates were resuspended in 100 μl of 1× SDS loading buffer, and 10 μl samples were subjected to SDS-PAGE analysis. The amount of secreted rHtA was determined by gel scanning as described. The wet weight of cultured cells was obtained by centrifugation. Protein concentration in the supernatant was determined by Bradford assay. The expressed rHtA was verified by western blot using a rabbit anti-His×6 IgG antibody.

### Purification of rHtA

After an 84-h induction with methanol, 300 ml of the supernatant from the bioreactor was centrifuged at 15,000*g* for 60 min at 4 °C, followed by adjustment to pH 8.0 with 2 M sodium hydroxide. Using a peristaltic pump at a flow rate of 1.5 ml/min, the supernatant was loaded onto a 50-ml column containing 4 ml Ni^+^-NTA resin (Qiagen, Guangzhou, China) that was pre-equilibrated with buffer A (20 mM PBS, pH 8.0, and 200 mM NaCl). The column was then washed with 200 ml buffer B (20 mM PBS, pH 8.0, 200 mM NaCl, and 30 mM imidazole) to remove unbound proteins. rHtA was eluted using an imidazole gradient ranging from 50 to 500 mM imidazole in 20 ml buffer B. The collected protein samples were subjected to SDS-PAGE analysis and stained with Coomassie Brilliant Blue R250.

All collected protein samples were concentrated using 15-ml filters with a molecular weight cut off of 3 kDa (Millipore, Billerica, MA, USA), centrifuged at 4000*g* for 60 min at 4 °C, subjected to SDS-PAGE analysis and stained with Coomassie Brilliant Blue R250. The concentrated proteins were dialyzed against 500 ml buffer C (40 mM PBS, pH 8.5, and 50 mM NaCl) at 4 °C three times. After centrifugation at 15,000*g* for 60 min at 4 °C, the dialyzed supernatants were loaded onto a 1-ml CM Sepharose FF column (GE Health care, Guangzhou, China) that was pre-equilibrated using buffer C. The column was washed with 400 ml buffer D (40 mM PBS, pH 8.5, and 100 mM NaCl) to remove unbound proteins. rHtA was eluted with buffer E (40 mM PBS, pH 7.4, and 500 mM NaCl). After dialysis against 40 mM PBS, pH 7.4, and 50 mM NaCl, the collected solution was concentrated and subjected to SDS-PAGE analysis in the presence or absence of DTT and staining with Coomassie Brilliant Blue R250.

### Determination of the molecular weight (MW) and amino acid sequence of rHtA

The MW of rHtA was determined by high-performance liquid chromatography using 30 μg of purified rHtA, a C4 chromatographic column (Agilent Technologies, Santa Clara, CA, USA), and a high resolution 6520 quadrupole time of flight mass spectrometry (Q-TOF–MS) proteomics analyser (Agilent Technologies) with a scan range from 500 to 4000 m/z. The accurate MW of rHtA was calculated by deconvolution of the raw MS data.

The amino acid sequence of rHtA was determined by mass spectrometry. The purified rHtA was reduced for 40 min with 5 mM DTT at room temperature followed by alkylation for 40 min with 15 mM iodoacetamide in a dark room. The alkylated protein samples were digested overnight at 37 °C with trypsin (Promega, Shanghai, China) at a 1:50 enzyme-to-substrate ratio. Following digestion, the peptide mixtures were acidified with trifluoroacetic acid to 1% and desalted. The desalted peptide samples were dried in a vacuum concentrator, dissolved in 10 μl of 0.1% formic acid in water, and the purified peptide samples were subjected to nano-liquid chromatography tandem MS (nano-LC–MS/MS) analysis. Raw MS data were analysed and searched against protein-sequence databases using Proteome Discoverer (Thermo Fisher Scientific, Waltham, MA, USA).

### Thermal- and protease-stability analyses

Thermal-stability analysis was determined by incubation of 100 μl of 1 mg/ml pure rHtA in PBS for 0, 0.5, 1, 2, and 4 h at 98 °C. After centrifugation at 17,000*g* for 30 min at 4 °C, 40 μl of the supernatant was mixed with 10 μl of 5× SDS loading buffer, and 10-μl samples were subjected to SDS-PAGE analysis. Protease stability was determined by incubating 1 mg/ml pure rHtA in PBS for 0, 15, 30, 60, and 120 min at 30 °C with proteinase K or trypsin at a final concentration of 500 μg/ml. At the end of the incubation period, 3 mM phenylmethanesulfonyl fluoride was added to terminate the reaction. Finally, 20 μl of the digestion products were mixed with 5 μl of 5× SDS loading buffer, and 10-μl samples were subjected to SDS-PAGE analysis.

### MTT assay

Sf9 cells (Procell, Wuhan, China) were cultured in insect-cell medium (Sino Biological, Beijing, China) in a cell-culture flask at 27 °C. When the cell confluence reached 80–90%, cells were resuspended in insect-cell medium, and the cell density was adjusted to 2 × 10^5^ cells/ml. Sf9 cells (100 μl) were added to each well of a 96-well plate (Corning, Guangzhou, China) and incubated at 27 °C for 24 h. Different final concentrations of rHtA (0, 1, 2, 4, 8, 16, 32, and 64 ng/ml) were added and incubated at 27 °C for 48 h, and cell proliferation was determined by 3-(4,5-dimethylthiazol-2-yl)-2,5-diphenyltetrazolium bromide (MTT) assay. Briefly, 10 μl MTT solution (5 mg/ml MTT in PBS) was added to each well of the 96-well plate, followed by incubation at 27 °C for 4 h, after which media supernatants were discarded carefully. Dimethyl sulfoxide (100 μl) was then added to each well to dissolve the formazan for 10 min at 37 °C. Absorbance was measured on an ELISA plate reader (BioTek ELX800, BioTek Instruments Inc., Vermont, USA) at a wavelength of 570 nm.

### Insect bioassays

To evaluate the injection bioactivity of purified rHtA, the final instar larval-stage *G. mellonella* were used (weight: 0.22 ± 0.04 g). *G. mellonella* were incubated on ice prior to use. Recombinant HtA was injected at the third and fourth abdominal segments using 10-μl sharp Hamilton syringes (Sigma-Aldrich, St. Louis, MO, USA). Each larva was injected with 1, 2, 4 or 8 μg of purified rHtA (1 mg/ml in PBS) or 10 μl PBS as control. Post-injection toxicity was monitored with a lethality test every 24 h, and dead larvae were immediately removed. For each treatment, three replicates of 30 larvae were tested.

## Additional file

**Additional file 1.** Video after five days of injection 1 μg of rHtA per larva or PBS.

## Abbreviations

HRP: horseradish peroxidase
SDS-PAGE: sodium dodecyl sulfate-polyacrylamide gel electrophoresis
NTA: Ni^2+^-nitriloacetic acid
MWCO: molecular weight cut off
TMS: trace metal solution
PCR: polymerase chain reaction
MW: molecular weight
DO: dissolved oxygen
PBS: phosphate buffered solution
PMSF: phenylmethanesulfonyl fluoride
DTT: DL-Dithiothreitol
MTT: 3-(4, 5-dimethylthiazol-2-yl)-2, 5-diphenyltetrazolium bromide
